# A novel nested polymerase chain reaction targeting the testis-specific protein Y-encoded family of genes for high sensitivity of recent semen exposure detection: Comparison with four other assays of semen detection

**DOI:** 10.1371/journal.pone.0220326

**Published:** 2019-07-25

**Authors:** Katia Giguère, François A. Leblond, Ella Goma-Matsétsé, Vibhuti Dave, Luc Béhanzin, Fernand A. Guédou, Michel Alary

**Affiliations:** 1 Centre de recherche du CHU de Québec, Université Laval, Québec, Québec, Canada; 2 Département de médecine sociale et préventive, Université Laval, Québec, Québec, Canada; 3 Centre de Recherche de l’Hôpital Maisonneuve Rosemont, Montréal, Québec, Canada; 4 Dispensaire IST, Centre de santé communal de Cotonou 1, Cotonou, Bénin; 5 École Nationale de Formation des Techniciens Supérieurs en Santé Publique et en Surveillance Épidémiologique, Université de Parakou, Parakou, Bénin; 6 Institut national de santé publique du Québec, Québec, Québec, Canada; Massachusetts General Hospital, UNITED STATES

## Abstract

**Objectives:**

Because self-report of sexual behaviours is prone to biases, biomarkers of recent semen exposure are increasingly used to assess unprotected sex. We aimed to present a novel nested polymerase chain reaction (PCR) assay targeting testis-specific protein Y-encoded (*TSPY*) genes and to compare its performance in detecting recent semen exposure with that of four other assays.

**Methods:**

Forty-five vaginal samples were selected at baseline of a prospective observational demonstration study of early antiretroviral treatment and pre-exposure prophylaxis among female sex workers in Benin. Semen exposure was assessed with: a rapid prostate-specific antigen (PSA) detection assay, a quantitative PCR targeting the sex-determining region (*SRY*) gene, a standard PCR targeting *SRY*, a standard PCR targeting *TSPY*, and a nested PCR targeting *TSPY* (n-TSPY). Because we had hypothesized that n-TSPY would be the most sensitive of the five assays while remaining specific, and as our results suggested that it was the case, sensitivity and specificity were calculated for each assay in comparison with n-TSPY.

**Results:**

The n-TSPY could detect male DNA at concentration 16 and 64 times lower compared to s-TSPY and s-SRY, respectively. Among the 45 vaginal samples, prevalences of semen exposure according to the different assays varied from 22.2% (95%CI: 11.2%-37.1%) to 70.5% (95%CI: 54.8%-83.2%), with the highest prevalence measured with n-TSPY. The n-TSPY products were of expected size and we observed no false-positive in female DNA controls. The assay that offered the second best performance in detecting semen exposure was the PSA rapid test, with a sensitivity of 61.3% and a specificity of 100% compared to n-TSPY.

**Conclusions:**

Compared to n-TSPY, all other PCR assays had poor performance to detect semen exposure. The n-TSPY is an accessible assay that may have great utility in assessing semen exposure in studies where many factors are expected to accelerate biomarkers’ clearance.

## Introduction

Unprotected sex being a major risk factor for human immunodeficiency virus (HIV) and other sexually transmitted infections (STI), it must be accurately measured in HIV/STI surveillance, treatment, and prevention research. Although the most common method used to monitor condom use is self-report, this method is prone to social desirability and recall biases [1]. To circumvent those biases, biomarkers of recent semen exposure have been used to detect unprotected sex among women. Among the most characterized and used biomarkers are the prostate-specific antigen (PSA) and Y-chromosomal deoxyribonucleic acid (Yc-DNA) [2].

PSA is a protein that can be found at mean concentrations >1 mg/ml in seminal fluid of healthy men [3] and that can be detected in vaginal samples for a mean period of 20 to 27 hours and up to two days after exposure to semen [4–8]. Yc-DNA is unique to males and is found in every cells of a man, including spermatozoa. Using a polymerase chain reaction (PCR), Yc-DNA can be detected in vaginal samples up to two weeks after exposure to semen with a half-life for clearance of 3.8 days [9, 10].

The most commonly targeted gene to detect Yc-DNA by the use of PCR is the sex-determining region Y (*SRY*) gene [11]. Most recently, the testis-specific protein Y-encoded (*TSPY*) family of homologous genes has been identified as a novel target to detect Yc-DNA in vaginal samples [12]. Both standard PCR followed by gel electrophoresis detection and quantitative PCR have been used to detect *SRY* and *TSPY* genes in vaginal samples. With the use of either a standard or a quantitative PCR, *TSPY* genes have been detected at higher rates and over a longer period of time than *SRY* in vaginal samples [12, 13], while the use of a quantitative PCR increased sensitivity and window of detection of both *SRY* and *TSPY* genes compared to the use of a standard PCR [13].

Other factors were shown to affect the sensitivity of Yc-DNA detection. Vaginal inoculation with low amount of semen has led to lower sensitivity of Yc-DNA detection compared to inoculation with higher amounts of semen [4]. Post-coital Yc-DNA levels were shown to be lower during menses compared to non-menses periods [14]. Vaginal douching, a highly prevalent feminine hygiene practice [15], is expected to accelerate the biomarkers’ clearance. *In vitro* assays have shown that some microbicides and lubricants could lead to partial or total inhibition of Yc-DNA detection [16, 17]. In observational settings where time since semen exposure and thus, biomarkers concentration, may vary, where condom breakage or slippage may lead to low exposure to semen, where menses or vaginal douching are expected to washout semen, and where women can use topical products that might interfere with biomarkers detection, an assay that allows the detection of low target concentrations is required.

Given the observations above, a quantitative PCR targeting *TSPY* genes might seem the best option to detect unprotected sex in observational settings. However, quantitative PCR requires specialized and expensive equipment that is not always available in settings such as low- and middle-income countries. Nested PCR is a more easily accessible method that consists of two successive rounds of standard PCR with each round using different sets of primers. The double rounds of amplification increase the sensitivity of the assay while the double sets of primers increase its specificity [18].

In the context of a prospective observational demonstration study aiming to assess the feasibility and usefulness of early antiretroviral treatment (E-ART) and pre-exposure prophylaxis (PrEP) among female sex workers (FSW) in Cotonou, Benin [19], we hypothesized that a nested PCR targeting *TSPY* would be more sensitive than standard or quantitative PCR assays targeting *SRY* or *TSPY* to detect semen exposure while remaining specific. In this article, we present a novel nested PCR assay targeting *TSPY* genes (n-TSPY) and compare its performance in detecting semen exposure with that of four other assays: a rapid PSA detection test (PSA), a commercial quantitative PCR targeting *SRY* (q-SRY), a standard PCR targeting *SRY* (s-SRY), and a standard PCR targeting *TSPY* (s-TSPY).

## Materials and methods

Vaginal specimens analysed in this study were collected with cotton swabs by a physician at baseline of a larger prospective observational demonstration study that aimed to assess the feasibility and usefulness of E-ART and PrEP among FSW in Cotonou, Benin [19]. This study was conducted from October 2014 to December 2016. At baseline, a face-to-face interview was administered to the participants in a private setting by two trained interviewers to assess socio-demographic characteristics and sexual behaviours from the past 2 and 14 days. For each of the two recall periods, participants who reported vaginal sex were classified as having reported unprotected sex if condoms were not always used and/or if an episode of condom breakage or slippage occurred in the recall period.

At recruitment, participants provided written informed consent for E-ART/PrEP study participation but were not informed of the specific purpose of PSA and Yc-DNA detection until the end of the study (December 2016) to limit information bias on self-report of unprotected sex. Participants were free to decline or withdraw from the study at any time. The protocol, including procedures for delayed information, was approved by the Benin National Ethics Committee for Health Research and the ethics committee of CHU de Québec—Université Laval.

### Sample selection

As we hypothesised that the n-TSPY would be the most sensitive of the five recent semen exposure assays, we planned to compare the different assays to the former one. To better define the capacity of the different assays to detect semen exposure compared to the n-TSPY, we aimed to select vaginal samples of different Yc-DNA concentration levels. We thus first performed a pilot study to estimate the prevalence of Yc-DNA positive samples as well as to qualitatively quantify those samples. Briefly, we tested vaginal samples of the first 196 FSW recruited in the E-ART/PrEP study in one replicate using n-TSPY. The n-TSPY assay procedures are described below (see Detection of Y-chromosomal deoxyribonucleic acid). Since the prevalence of positive samples was estimated at 29%, we randomly selected 30 samples among the 361 recruited FSW, expecting to have about 21 negative samples ((1–0.29) x 30) for the calculation of specificities. To ensure a variety of positive samples for the calculation of sensitivities, we randomly selected 15 more positive samples as follow: 5 low-, 5 moderate-, and 5 high-positive n-TSPY samples. Since the samples were run in replicates of one, the qualitative quantification was based on a visual examination of the intensity of the migration bands on agarose gel.

### Sample preparation

After collection by a clinician, vaginal swabs were stored at -20°C for a maximum of one week before extraction of PSA and total DNA. To extract PSA, we eluted each vaginal swab for two hours at 4°C in an extraction solution provided in the ABAcard p30 kit (Abacus Diagnostics, West Hills, CA), a commercially available rapid immunodetection test for PSA. After incubation, we gently pressed the swab on the inner wall of the tube to recover the maximum amount of solution and we centrifuged the extract at 10,000 x g for 5 min at room temperature. We then recovered and stored the supernatant at -20°C for a maximum of one week before PSA testing. We extracted total DNA from the remaining cellular pellet using the commercially available QIAamp DNA extraction kit (Qiagen AG, Basel, Switzerland) according to the manufacturer’s instructions. We stored the total DNA extract (about 210 μl) at -80°C for a maximum of two weeks before Yc-DNA testing except for testing with q-SRY (maximum of 9 months).

### Detection of prostate-specific antigen

We detected PSA using ABAcard p30 (Abacus Diagnostics, West Hills, CA) according to the manufacturer’s instructions. We chose ABAcard p30 because it is easy to use, is relatively inexpensive, has shown good performance compared to a quantitative assay [20], and is commonly used in studies assessing semen exposure [21–26]. Briefly, we added 200 μl of the extract solution to the sample well of a strip test and incubated the strip for 10 min at room temperature. A pink line both at the test (T) and control (C) positions indicated a positive result while a pink line at the C position only indicated a negative result. We observed no inconclusive result (i.e. absence of a pink line at the C position).

### Detection of Y-chromosomal deoxyribonucleic acid

We detected Yc-DNA by means of four different PCR assays: a commercial quantitative PCR targeting *SRY* (q-SRY), a standard PCR targeting *SRY* (s-SRY), a standard PCR targeting *TSPY* (s-TSPY), and a nested PCR targeting *TSPY* (n-TSPY). We performed q-SRY with the Quantifiler Duo DNA Quantification kit (Applied Biosystems, ThermoFisher Scientific, Waltham, MA) according to the manufacturer’s guidelines with no modification [27]. Briefly, we added 2 μl of total DNA extract to 23 μl of Quantifiler Duo Primer Mix. We performed amplification using a 7500 real-time PCR system (Applied Biosystems, ThermoFisher Scientific, Waltham, MA) with amplification settings reported in Table 1. Primers and probe sequences of the q-SRY being proprietary, this information could not be retrieved.

**Table 1:** Characteristics of the polymerase chain reaction assays.

Assay	Primers sequences	Amplicon length (bp)	Amplification settings
q-SRY	NA	130	50°C for 2 min, initial denaturation at 95°C for 10 min, followed by 40 cycles of amplification (95°C, 15 s; 60°C, 60 s)
s-SRY	F-5’-CGC ATT CAT CGT GTG GTC TCG-3’ R-5’-ATT CTT CGG CAG CAT CTT CGC-3’	229	Initial denaturation at 94°C for 4 min, followed by 35 cycles of amplification (94°C, 30 s; 64°C, 30 s; and 70°C, 60 s) and a final elongation for 10 min at 70°C
s-TSPY	F-5’-CGT CAT CCA GAG CGT CCC-3’ R-5’-TTT CCA CAG CCA CAC TGG TC-3’	178	Initial denaturation at 94°C for 4 min, followed by 35 cycles of amplification (94°C, 30 s; 63°C, 30 s; and 70°C, 60 s) and a final elongation for 10 min at 70°C
n-TSPY	F-5’-GGG CCA ATG TTG TAT CCT TCT C-3’ R-5’-GTT CCC CAA AGA GTC ACA TCG-3’	115	Initial denaturation at 94°C for 4 min, followed by 35 cycles of amplification (94°C, 30 s; 64°C, 30 s; and 70°C, 60 s) and a final elongation for 10 min at 70°C

PCR, polymerase chain reaction; NA, not available; bp, base pairs; *SRY*, sex-determining region Y gene; *TSPY*, testis-specific protein Y-encoded family of genes; q-SRY, quantitative PCR targeting *SRY*; s-SRY, standard PCR targeting *SRY*; s-TSPY, standard PCR targeting *TSPY*; n-TSPY, nested PCR targeting *TSPY*.

We synthesized primers for s-SRY according to previously published sequences [28]. We designed primers for s-TSPY and n-TSPY using Primer3 [29], and confirmed the specificity of each pair of primers with the National Center for Biotechnology Information’s Nucleotide Basic Local Alignment Search Tool (BLAST). Primers sequences are reported in Table 1.

We used EconoTaq Plus Green 2X Master Mix (Lucigen, Middleton, Wisconsin, USA) for amplifications with standard and nested PCR assays. For s-SRY and s-TSPY, we added 5 μl of total DNA extract to 20 μl of master mix containing 1 μM of primers and EconoTaq solution according to manufacturer instructions [30]. For n-TSPY, we added 5 μl of a 1:10 dilution of s-TSPY amplification products to 20 μl of master mix containing 1 μM of primers targeting *TSPY* inside the previously amplified sequence. We performed amplification using a Bio-Rad iCycler (Bio-Rad Laboratories, Hercules, CA, USA). Amplification settings are reported for each PCR assay in Table 1. After amplification, electrophoresis on a 2% agarose gel, and staining with Gel Red (Biotium Inc. Fremont, CA), we visualized the final PCR products of s-SRY (229 base pairs (bp)), s-TSPY (178 bp), and n-TSPY (115 bp) under ultraviolet transillumination.

We ran each test sample in replicates of five for q-SRY or in replicates of three for s-SRY, s-TSPY, and n-TSPY. A sample was considered negative if all replicates had no amplification and positive if at least one replicate had amplification. For q-SRY, a sample was also considered quantifiable if at least three replicates of five had amplification [31]. The median concentration of Yc-DNA (copies/sample) was calculated among replicates of all quantifiable samples together.

In each PCR assay, we used DNA extracted from peripheral blood mononuclear cells from male and female donors as positive and negative controls, respectively, and we used nuclease-free water as no template control (NTC). All laboratory procedures were performed by female technicians to avoid male DNA contamination.

### Serial dilution assays of male deoxyribonucleic acid

We amplified two-fold dilutions of male DNA positive control, from non-diluted to 1:838,9 x 10^4^ diluted in nuclease-free water, by means of s-SRY, s-TSPY, and n-TSPY to assess the highest dilution to which each of these PCR could detect Yc-DNA.

### Statistical analyses

The proportion of women with semen exposure according to each of the five assays was calculated and reported with 95% confidence intervals (95%CI). As we had hypothesized that n-TSPY would be the most sensitive of the five assays while remaining specific, and as our results suggest that it was the case (see Results section), sensitivity and specificity were calculated for the four other individual assay compared to n-TSPY. To better define the capacity of the different assays to detect semen exposure compared to n-TSPY, we intended to calculate sensitivity among all n-TSPY positive samples, but also among low-, moderate-, or high-positive n-TSPY samples as defined by the number of replicates with amplification (one, two, or three out of three, respectively). However, due to the low number of moderate-positive n-TSPY samples (n = 2), these were pooled together with low-positive samples for the calculation of sensitivities by subgroups. We did not calculate positive and negative predictive values because these measures of validity are not intrinsic to the test itself but vary for given values of sensitivity and specificity as a function of the prevalence in the population.

Agreement of results between n-TSPY and each of the four other assays was tested using McNemar’s test. Statistical analyses were conducted using SAS Studio, version 3.71 (SAS Institute Inc., Cary, NC, USA).

## Results

### Serial dilution assays

**Fig 1:**
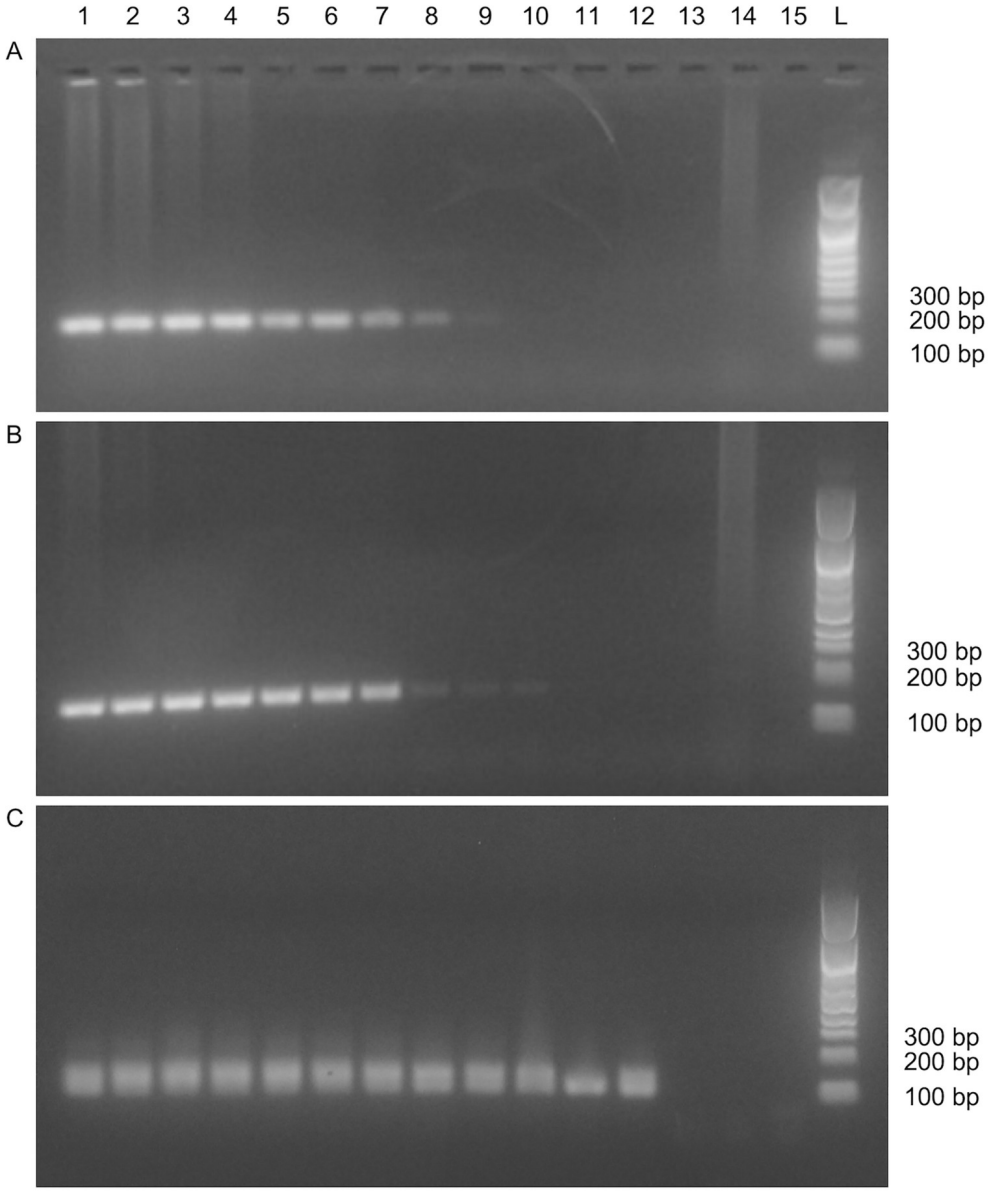
Agarose gel showing PCR amplification products from male DNA serial dilutions. Two-fold serial dilutions of male DNA were amplified by the means of (A) s-SRY, (B) s-TSPY, and (C) n-TSPY. Lane 1, no dilution; lanes 2 to 13 show one-in-two of the two-fold male DNA dilutions (from 1:2 to 1:838,9 x 10^4^); lane 14, female DNA; lane 15, no template control; lane L, 100–1500 bp DNA ladder (Bio Basic, Markham, Ontario, CA).

Fig 1 shows amplification of male DNA serial dilutions by the means of s-SRY, s-TSPY, and n-TSPY. The highest male DNA dilutions that could be detected was 1:3,3 x 10^4^ with s-SRY, 1:13,1 x 10^4^ with s-TSPY, and 1:209,7 x 10^4^ with n-TSPY. The s-TSPY could detect male DNA at concentration four times lower than s-SRY, and the n-TSPY, at concentration 16 and 64 times lower compared to s-TSPY and s-SRY, respectively.

### Prevalence of semen exposure

Out of the 361 FSW recruited in the E-ART/PrEP study, we randomly selected 30 vaginal samples, and, to ensure a variety of positive samples for the calculation of sensitivities, we randomly selected 15 more n-TSPY-positive samples, for a total of 45 samples from 45 different FSW. Socio-demographic characteristics as well as sexual behaviours from the last 2 and 14 days are reported in Table 2 for the 45 included women.

**Table 2:** Socio-demographic characteristics and behaviours at baseline (n = 45).

Variables	n	(%)
Age, mean (SD)	34.3	(9.3)
Nationality		
Beninese	21	(46.7)
Congolese	1	(2.2)
Ghanean	7	(15.6)
Nigerian	7	(15.6)
Togolese	9	(20.0)
Education		
None	17	(37.8)
Primary	14	(31.1)
Secondary	13	(22.2)
University	1	(2.2)
Marital status		
Never married	14	(31.1)
Divorced/Separated	26	(57.8)
Widowed	4	(8.9)
Married	1	(2.2)
Contraceptive method (n = 44)		
None	26	(59.1)
Condom only	7	(15.9)
Hormonal	9	(20.5)
Traditional method	2	(4.5)
Had menses in the last 2 days (n = 25)*	2	(8.0)
Had menses in the last 14 days (n = 25)*	10	(40.0)
Behaviours in the last 2 days		
Had at least one vaginal douche (n = 28)*	28	(100.0)
Had vaginal sex	35	(77.8)
Had vaginal sex without a condom	9	(20.0)
Experienced condom breakage or slippage	3	(6.7)
Had unprotected sex (sex without condom or with condom breakage or slippage) (n = 44)	12	(27.3)
Behaviours in the last 14 days		
Had at least one vaginal douche (n = 28)*	28	(100.0)
Had vaginal sex	42	(93.3)
Had vaginal sex without a condom (n = 44)	16	(36.4)
Experienced condom breakage or slippage (n = 41)	7	(17.1)
Had unprotected sex (sex without condom or with condom breakage or slippage) (n = 43)	20	(46.5)

SD, standard deviation.

* Seventeen participants have missing data for menses and vaginal douching because of the late introduction of questions on menses and vaginal douching practices after the beginning of the recruitment in the E-ART/PrEP study.

The subset of 45 vaginal samples were tested for semen exposure by means of PSA, q-SRY, s-SRY, s-TSPY, and n-TSPY. Two women had missing data for at least one of the assays. Thus, the proportion of women with semen exposure was calculated among all available data for each assay while comparison analyses were restricted to the 43 samples for which all five semen exposure measures were available. Prevalences of semen exposure according to the different assays varied from 22.2% (95%CI: 11.2%-37.1%) to 70.5% (95%CI: 54.8%-83.2%), with the lowest prevalence having been measured with s-SRY and the highest, with n-TSPY (Table 3).

**Table 3:** Prevalence of recent semen exposure according to the compared assays.

Assay	n	Semen exposure % (95%CI)	
PSA rapid test	19/45	42.2	(27.7–57.9)
q-SRY	14/45	31.1	(18.2–46.7)
Quantifiable ^a^	4/14	28.6	(8.4–58.1)
s-SRY	10/45	22.2	(11.2–37.1)
s-TSPY	13/43	30.2	(17.2–46.1)
n-TSPY ^b^	31/44	70.5	(54.8–83.2)
Low-positive	13/31	41.9	(24.6–60.9)
Moderate-positive	2/31	6.5	(0.8–21.4)
High-positive	16/31	51.6	(33.1–69.9)

PSA, prostate-specific antigen; *SRY*, sex-determining region Y gene; *TSPY*, testis-specific protein Y-encoded family of genes; q-SRY, quantitative PCR targeting *SRY*; s-SRY, standard PCR targeting *SRY*; s-TSPY, standard PCR targeting *TSPY*; n-TSPY, nested PCR targeting *TSPY*.

^a^ A test was considered quantifiable when at least three of five replicates had amplification.

^b^ A test was considered low-, moderate-, or high-positive when one, two, or three replicates out of three had amplification.

A total of 14 samples were positive with q-SRY (31.1%; 95%CI: 18.2%-46.7%). Out of these, four samples (28.6%; 95%CI: 8.4–58.1%) were quantifiable, with a median concentration of 170 Yc-DNA copies/sample (IQR = 97–319 copies/sample).

**Fig 2:**
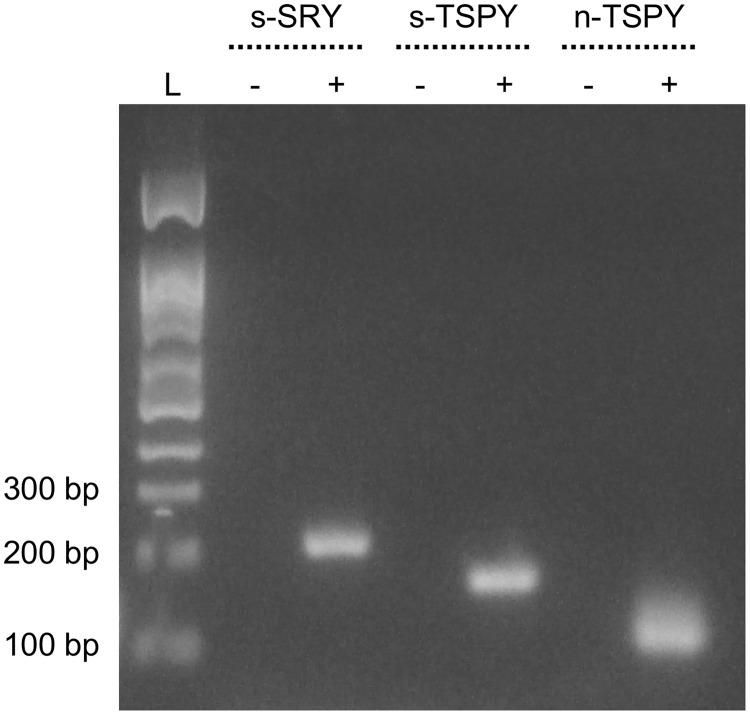
Agarose gel showing standard and nested PCR products size. Female (-) and male (+) DNA extracted from peripheral blood mononuclear cells were amplified with a standard PCR targeting *SRY* (s-SRY), a standard PCR targeting *TSPY* (s-TSPY), and a nested PCR targeting *TSPY* (n-TSPY), respectively. Lane L, 100–1500 bp DNA ladder (Bio Basic, Markham, Ontario, CA).

None of the PSA tests was inconclusive. No case of false-positive Yc-DNA was observed in any of the negative or no template controls that were run with any of the PCR assays. All standard and nested PCR products were of expected size. Fig 2 presents an agarose gel showing s-SRY, s-TSPY, and n-TSPY PCR products sizes following amplification of female and male DNA.

### Performance of four assays of semen exposure compared to the n-TSPY assay

Among the 43 samples for which all five semen exposure measurements were available, 31 (72.1%) were tested positive with n-TSPY. Of those 31 positive samples, 13 were low-, two were moderate-, and 16 were high-positive samples. Due to the low number of moderate-positive samples (n = 2), these were pooled together with low-positive samples for the calculation of sensitivities by subgroups.

Sensitivity, specificity, and McNemar’s tests results for each assay compared to n-TSPY are reported in Table 4. Compared to n-TSPY, the proportion of positivity was lower for all the four other assays (p<0.001, McNemar’s test). When calculated among all n-TSPY positive samples, the sensitivity of the four other assays varied from 29.0% (95%CI: 14.2%-48.0%) to 61.3% (95%IC: 42.2%-78.2%), with s-SRY having the lowest sensitivity and PSA having the highest sensitivity to detect semen among positive n-TSPY samples. The s-TSPY (41.9%; 95%CI: 24.6%-60.9%) was more sensitive than s-SRY (29.0%; 95%CI: 14.2%-48.0%) to detect Yc-DNA, while q-SRY (45.2%; 95%CI: 27.3%-64.0%) was more sensitive than either s-SRY or s-TSPY to detect Yc-DNA. Despite showing the second highest sensitivity, the q-SRY could only quantify 12.9% (95%IC: 3.6%-29.8%) of the 31 n-TSPY positive samples.

**Table 4:** Performance of four assays of semen exposure compared to the nested TSPY PCR assay (n = 43).

	Sensitivity (95%, CI) ^a^						Specificity (95%CI)		p-value ^b^
Assay	All positive		Low/moderate-positive		High-positive				
	(n = 31)		(n = 15)		(n = 16)				
PSA rapid test	61.3	(42.2–78.2)	26.7	(7.8–55.1)	93.8	(69.8–99.8)	100.0	(73.5–100.0)	<0.001
q-SRY									
Positive	45.2	(27.3–64.0)	0.0	(NA)	87.5	(61.7–98.5)	100.0	(73.5–100.0)	<0.001
Quantifiable ^c^	12.9	(3.6–29.8)	0.0	(NA)	25.0	(7.3–52.4)	100.0	(73.5–100.0)	<0.001
s-SRY	29.0	(14.2–48.0)	6.7	(0.2–32.0)	50.0	(24.7–75.4)	91.7	(61.5–99.8)	<0.001
s-TSPY	41.9	(24.6–60.9)	6.7	(0.2–32.0)	75.0	(47.6–92.7)	100.0	(73.5–100.0)	<0.001

NA, not available; PCR, polymerase chain reaction; PSA, prostate-specific antigen; q-SRY, quantitative PCR targeting the sex-determining region Y gene; s-SRY, standard PCR targeting the sex-determining region Y gene; s-TSPY, standard PCR targeting the testis-specific protein Y-encoded family of genes; n-TSPY, nested PCR targeting the testis-specific protein Y-encoded family of genes.

^a^ Sensitivity was calculated among all n-TSPY positive samples (at least one replicate out of three with amplification) and among low/moderate- (one/two replicates out of three with amplification) or high-positive (three replicates out of three with amplification) n-TSPY positive samples.

^b^ A McNemar’s test was used to evaluate the agreement of results between n-TSPY and each of the four other tests.

^c^ A test was considered quantifiable when at least three of five replicates had amplification.

When calculated among low/moderate-positive n-TSPY, sensitivity was very low for all assays. The q-SRY could detect none of the 15 low/moderate-positive samples while the highest sensitivity was estimated at only 26.7% (95%CI: 7.8%-55.1%), with PSA. Among high-positive samples, sensitivity was higher for all assays. The s-SRY had the lowest sensitivity with 50.0% (95%CI: 24.7%-75.4%). The q-SRY could detect 87.5% (95%CI: 61.7%-98.5%) of the 16 high-positive samples, but could only quantify 25.0% (95%CI: 7.3%-52.4%) of these.

## Discussion

We presented a novel nested PCR assay targeting *TSPY* genes and compared its performance in detecting semen exposure with that of four other assays: a rapid PSA detection test, a commercial quantitative PCR targeting *SRY*, a standard PCR targeting *SRY*, and a standard PCR targeting *TSPY*.

We first performed a male DNA serial dilution assay to assess the highest dilution to which s-SRY, s-TSPY, and n-TSPY could detect Yc-DNA. We observed that s-TSPY could detect male DNA at a concentration four times lower than s-SRY, which is in agreement with previous studies having shown that *TSPY* genes are more sensitive than *SRY* to detect Yc-DNA [12, 13]. Testis-specific protein Y-encoded being a family of homologous genes, multiple copies of *TSPY* are present on a single Y-chromosome. Therefore, targeting *TSPY* allows amplification of Yc-DNA to a higher extent than targeting *SRY* that is present in only one copy on Y-chromosome, which in turn facilitates Yc-DNA detection [12]. We also observed that n-TSPY could detect male DNA at concentrations 16 and 64 times lower compared to s-TSPY and s-SRY, respectively. Those results confirmed our assumption that a nested PCR targeting *TSPY* would be more sensitive than standard PCR targeting either *SRY* or *TSPY* to detect male DNA. Compared to standard PCR, the number of amplification cycles is greatly increased with nested PCR due to the double rounds of amplification, which thereby increases the sensitivity of detection [18].

Among a subset of 45 women at baseline of the E-ART/PrEP study, the highest prevalence of semen exposure was measured with n-TSPY, which suggests that n-TSPY was more sensitive than any of the four other assays. This can only be true if the supplemental positive cases detected with n-TSPY were not false-positive results. Although we cannot completely exclude the possibility of false-positive results using n-TSPY, especially when considering the low-positive results, different factors suggest that false n-TSPY positive results are unlikely in our study. First, compared to a standard PCR, a nested PCR is expected to have higher specificity, or at least equivalent specificity if there is no margin to an increase in specificity, due to the use of two separate pairs of specific primers for the same target template [18]. Second, all s-TSPY and n-TSPY PCR products were of expected size, suggesting that primer pairs used for each of the two rounds of amplification for the nested PCR were specific to the targeted DNA sequence of the *TSPY* family of genes. Third, if the n-TSPY would have been unspecific, we would have expected to observe at least a few amplifications in the no template and/or negative controls. Not only none of the no template and negative controls has shown amplification when testing the 45 samples with n-TSPY, but no amplification was observed in any of the no template (n = 31) and negative controls (n = 31) when testing the 361 FSW at baseline of the E-ART/PrEP study. Finally, cross-contamination by male DNA during the laboratory procedures is also unlikely because only female technicians were involved in DNA extraction and amplification processes.

Compared to n-TSPY, all other assays have shown sensitivities <62% to detect positive n-TSPY. Consistent with our male DNA serial dilution assay results, sensitivity of s-TSPY(41.9%) was higher compared to that of s-SRY (29.0%) in the detection of positive n-TSPY. We also observed higher sensitivity to detect n-TSPY positive samples with q-SRY (45.2%) compared to s-SRY, which is consistent with a previous study having shown that quantitative PCR is more sensitive than standard PCR to detect Yc-DNA [13].

Noticeably, after n-TSPY, the assay that offered the best performance in detecting semen exposure was the PSA rapid test, with a sensitivity of 61.3% and a specificity of 100%. Lower sensitivity of q-SRY, s-SRY, and s-TSPY compared to PSA is unexpected. Indeed, because PSA can only be detected up to two days after semen exposure while Yc-DNA can be detected up to 14 days [4, 5, 9, 10], assays targeting Yc-DNA should be more sensitive to detect recent semen exposure than an assay targeting PSA. This inconsistency could be explained by false-positive PSA results, if, for example and as previously discussed [32], some participants of the E-ART/PrEP study had used K-Y (K-Y Brand Jelly, Johnson & Johnson), a commercial lubricant that can induce false-positive results with ABAcard p30 [33]. If PSA-positive results were true-positive results, the lower sensitivity of q-SRY, s-SRY, and s-TSPY compared to PSA could be explained by the former assays lacking sensitivity to detect even very recent semen exposure.

The q-SRY, s-SRY, and s-TSPY sensitivities were particularly low to detect low/moderate-positive n-TSPY samples (≤6.7%) compared to high-positive n-TSPY samples (≤87.5%), which might imply that the detection of Yc-DNA by the former assays was impaired by low DNA target concentrations. Indeed, the quantification performed with q-SRY suggests that concentrations of Yc-DNA in our subset of samples were low. Using the q-SRY, only 28.6% positive samples were quantifiable (12.9% of the n-TSPY positive samples), with a median concentration of 170 Yc-DNA copies/sample. In a previous study using the same commercially available q-SRY assay among sexually active women participating in a microbicide trial, sample concentrations were more than twice those we observed [31, 34]. The difference could be explained by a higher frequency of vaginal douching in our population where, at baseline, 100% of a random subset of 201 FSW reported at least one vaginal douche in the last two days [32], while only about 30% of participants reported having douched after sex in the microbicide trial [34]. It is also possible that quantitation with q-SRY was impaired in our study due to inhibitory products in vaginal samples. An in vitro experiment has previously shown that Replens, a commercial lubricant, inhibits the detection of Yc-DNA by quantitative PCR in a dose-dependent manner [17]. Though the inhibitory mechanism of Replens on quantitative PCR is unknown, it is also possible that Replens, if present in our samples, inhibited all of our PCR assays. In such context, n-TSPY could have had a better capacity to compensate an inhibitory effect than other PCR assays due to its lower threshold of detection.

This study is limited by a small sample size that yielded low statistical precision. Also, we could not assess time trends in Yc-DNA detection with n-TSPY following semen exposure because we cannot know for sure when semen exposure took place in our study. Longitudinal assessment of Yc-DNA detection using n-TSPY following semen exposure is needed to gain information on the window of detection of semen exposure. A limitation of n-TSPY is that, though more easily accessible than quantitative PCR, it still requires relatively specialized laboratory space, equipment, and trained staff as compared to rapid tests such as PSA.

Nonetheless, the relative performance of the different assays remains valid and still suggest that the n-TSPY assay would be the most performant for screening of recent semen exposure in observational settings. Strengths of this study include the comparison of a high number of assays for semen exposure with a novel Yc-DNA detection assay. Also, the compared assays were diversified, testing for semen exposure over different periods of time, targeting a protein or different genes, being quantitative or dichotomous, and using different types of technologies. Another strength of this study is to have compared the different assays in an observational setting where multiple factors such as time since semen exposure, amount of exposure, menses, vaginal douching, microbicides and lubricants use may have affected the biomarkers detection. Moreover, because all assays were performed using the same vaginal sample, confounding bias is avoided and the risk of observing differences due to variations in swab sampling is limited.

## Conclusions

Compared to n-TSPY, all other PCR assays had poor performance to detect semen exposure. The n-TSPY is an accessible assay that may have great utility to assess semen exposure in observational settings where multiple factors are expected to accelerate semen clearance, and in low- and middle-income settings where the need for objective assessment of unprotected sex is growing but where specialized equipment is not always available and affordable. PSA may still be suited for detection of very recent unprotected sex in low-technical environments with no access to PCR facilities. Further studies should assess time trends in detection of Yc-DNA with n-TSPY following semen exposure, impact of vaginal douching on semen detection with different assays, and impact of topical vaginal products on biomarkers detection in observational settings.

## Supporting information

S1 Table Database. This file contains the minimal anonymized data set necessary to replicate the study findings.
(XLSX)

S2 Table Codebook. This file contains all codes related to the anonymized data set file.
(DOCX)
