# Supplementary material for: A novel nested polymerase chain reaction targeting the testis-specific protein Y-encoded family of genes for high sensitivity of recent semen exposure detection: Comparison with four other assays of semen detection
Source: PLoS One. 2019 Jul 25;14(7):e0220326. doi: 10.1371/journal.pone.0220326 (PMC6657882; doi:10.1371/journal.pone.0220326)
Supplement: S2 Table — This file contains all codes related to the anonymized data set file. (DOCX) [file pone.0220326.s002.docx]

**S2 Table. Codebook**. Codes related to the anonymized data set file

| **Variable** | **Description** | **Code** |
| --- | --- | --- |
| IN | Subject identification number | Numeric |
| Age | Age in years | Numeric |
| Nation | Nationality | 0 = Beninese |
|  |  | 1 = Congolese |
|  |  | 2 = Ghanean |
|  |  | 3 = Nigerian |
|  |  | 4 = Togolese |
| Educ | Highest education level achieved | 0 = none |
|  |  | 1 = primary |
|  |  | 2 = secondary |
|  |  | 3 = university |
| Marital | Marital status | 0 = never married |
|  |  | 1 = divorced/separated |
|  |  | 2 = widowed |
|  |  | 3 = married |
| Contra | Contraception use | 0 = none |
|  |  | 1 = condom only |
|  |  | 2 = hormonal |
|  |  | 3 = traditional method |
| Menses2 | Had menses in the last 2 days | 0 = no |
|  |  | 1 = yes |
| Douche2 | Had at least one vaginal douche in the last 2 days | 0 = no |
|  |  | 1 = yes |
| Sex2 | Had vaginal sex in the last 2 days | 0 = no |
|  |  | 1 = yes |
| No_condom2 | Had vaginal sex without a condom in the last 2 days | 0 = no |
|  |  | 1 = yes |
| Break2 | Experienced condom breakage or slippage in the last 2 days | 0 = no |
|  |  | 1 = yes |
| Unprotected2 | Had vaginal sex without a condom or with condom breakage or slippage in the last 2 days | 0 = no |
|  |  | 1 = yes |
| Menses14 | Had menses in the last 14 days | 0 = no |
|  |  | 1 = yes |
| Douche14 | Had at least one vaginal douche in the last 14 days | 0 = no |
|  |  | 1 = yes |
| Sex14 | Had vaginal sex in the last 14 days | 0 = no |
|  |  | 1 = yes |
| No_condom14 | Had vaginal sex without a condom in the last 14 days | 0 = no |
|  |  | 1 = yes |
| Break14 | Experienced condom breakage or slippage in the last 14 days | 0 = no |
|  |  | 1 = yes |
| Unprotected14 | Had vaginal sex without a condom or with condom breakage or slippage in the last 14 days | 0 = no |
|  |  | 1 = yes |
| PSA | Testing for PSA | 0 = negative |
|  |  | 1 = positive |
| q_SRY_R1 | Yc-DNA copies/reaction (2 µl) in replicate 1 of q-SRY | Numeric |
| q_SRY_R2 | Yc-DNA copies/reaction (2 µl) in replicate 2 of q-SRY | Numeric |
| q_SRY_R3 | Yc-DNA copies/reaction (2 µl) in replicate 3 of q-SRY | Numeric |
| q_SRY_R4 | Yc-DNA copies/reaction (2 µl) in replicate 4 of q-SRY | Numeric |
| q_SRY_R5 | Yc-DNA copies/reaction (2 µl) in replicate 5 of q-SRY | Numeric |
| q_SRY | Testing for Yc-DNA with q-SRY | 0 = negative |
| q_SRY_quant | Quantifiability of sample with q-SRY | 0 = not quantifiable (<3 positive-replicates) |
|  |  | 1 = quantifiable (≥3 positive-replicates) |
| s_SRY | Testing for Yc-DNA with s-SRY | 0 = negative |
|  |  | 1 = positive |
| s_TSPY | Testing for Yc-DNA with s-TSPY | 0 = negative |
|  |  | 1 = positive |
| n_TSPY | Testing for Yc-DNA with n-TSPY | 0 = negative |
|  |  | 1 = positive |
| n_TSPY_cat | Testing for Yc-DNA with n-TSPY (positivity levels) | 0 = negative (0 positive-replicate) |
|  |  | 1 = low-positive (1 positive-replicate) |
|  |  | 2 = moderate-positive (2 positive-replicates) |
|  |  | 3 = high-positive (3 positive-replicates) |

Legend: PSA, prostate-specific antigen; Yc-DNA, Y chromosomal DNA; PCR, polymerase chain reaction; *SRY*, sex-determining region Y gene; *TSPY*, testis-specific protein Y-encoded family of genes; q-SRY, quantitative PCR targeting *SRY* gene; s-SRY, standard PCR targeting *SRY* gene; s-TSPY, standard PCR targeting *TSPY*; n-TSPY, nested PCR targeting *TSPY*
